# Johnson County Medical Society

**Published:** 1893-09

**Authors:** T. C. Osborn

**Affiliations:** Secretary; Cleburne, Texas


### Society Notes.

### JOHNSON COUNTY MEDICAL SOCIETY.

### INTERESTING AUTOPSY.

CLEBURNE, TEXAS, August 10, 1893.

A meeting of the Johnson County Medical Society was held to-day in the office of Dr. Alexander, with the President, Dr. J. L. Wagley, in the chair; and there were present as members Drs. Alexander, J. D. Osborn, T. C. Osborn, J. P. Sharp, J. L. Wagley, E. M. Williamson, of George's Creek P. O., Somerville Co., and J. J. Williamson of Cleburne.

The minutes of the meeting of July were read and approved.

The Board of Censors had no report to make.

Neither of the regular essayists were present and were continued over for the next meeting, and Drs. Evans, of George's Creek, and Culpepper, of Grandview, were added to read papers at the same meeting,

Dr. J. L. Wagley reported an interesting necroscopy in this way: A negro man named Ferguson had, last winter, an attack of pneumonia, from the result of which he died in July. I saw the case for the first time two weeks before his death. At that time he was laboring under great dispnœa, almost incessant cough, dullness over the left side of the chest, tenderness to pressure over the chest and epigastrium, prominence over the enciform cartilage of so decided a character as to induce me to believe that there was a purulent accumulation in the anterior mediastinum and to prepare for trephining the cavity; but the gravity of the symptoms forbade the operation at that time. Percussing the chest caused him to scream with pain; there was slight fever and much emaciation. I prescribed apyretics and alteratives with the ammonio-iodides in the lead, being satisfied there was effusion in both cavities of the chest. His death occurred in 12 hours after my visit. Permission was granted for only a partial post mortem examination. I found the mediastinum, heart and great blood vessels normal; the right pleural cavity free from fluid accumulation, but a number of adhesions existed between the surfaces, and three inches of hepatization in the lower border of the lung; left pleural cavity filled with serum, and no adhesion, but perfect collapse of the lung. The abdominal cavity was only partially explored. Found traces of hepatitis on the borders of the liver with adhesions to the stomach, and a low grade of peritonitis with thickening and induration of the mesentery, and bands of adhesion between the transverse colon and omentum. In other words, it was apparent that death was mainly caused by hepato-gastro-enteric lesions, notwithstanding the serious thoracic troubles manifested during life; and the history of pneumonia with dispnœa and constant and distressing cough thereafter, furnishes the main points of interest, namely: The multiplicity of expressions of disease exhibited by the chronic continuance of diseased conditions, as shown after death.

Dr. Wagley also reported a case of gunshot wound of the arm of a negro man, where the ball passed through the brachial artery, nerve and vein, and shattered the bone, but was not able to state more of the case as it was taken out of his care to a negro physician of Fort Worth, since which time he has not heard from the patient.

These cases were interestingly and courteously discussed by the members, and parallel cases were mentioned by several, but the principle in the discussion was in the treatment of accumulations of pus in the chest, whether to use disinfecting washes after the matter was let out. Drs. J. J. Williamson and T. C. Osborn defended the washing out as not only harmless, but actually necessary to hasten the cure, and they gave as an example the case of a man under their care where heated carbolized water was thrown freely into the cavity twice a day with a family syringe, and at every repetition the patient expressed decided relief.

On motion the meeting was adjourned until the 2nd Tuesday, the 13th day of September.

T. C. OSBORN, M. D., Secretary.

